# THE BENEFITS OF LONG-TERM CARE INSURANCE FOR FAMILY CAREGIVERS: EVIDENCE FROM CHINA

**DOI:** 10.1093/geroni/igad104.1516

**Published:** 2023-12-21

## Abstract

This paper aims to evaluate the impacts of the long-term care insurance (LTCI) program on family caregivers of older adults with functional limitations in mainland China. The study used data collected from 1,658 LTCI beneficiaries or those in the LTCI application process in 16 pilot Chinese cities in 2021. Descriptive statistics, chi-square tests, and multiple regression analyses were used to analyze the data. The results indicate that LTCI beneficiaries are more likely to receive institutional care than non-beneficiaries (27.8% vs. 16.8%), reducing the responsibilities and potential burden of family caregivers. LTCI beneficiaries are also more likely to receive social care services than non-beneficiaries (35.9% vs. 26.7%). Family caregivers of LTCI beneficiaries spend less time per day on care than non-beneficiaries (15.45 vs.16.59 hours), and they are less likely to delay their work due to caregiving (41.4% vs. 60.7%). Additionally, LTCI has a positive impact on various aspects for family caregivers, with over 40% reducing their caregiving time, 55.0% reducing care service costs, 68.5% feeling that LTCI has reduced their physical burden, and 77.9% indicating that their psychological burden has been lessened. The study concludes that the establishment of long-term care insurance nationwide should be promoted as the pilot sites have seen significant positive effects on family caregivers. LTCI should also consider more direct support to family caregivers.

